# Maximum Equilibrium Prevalence of Mosquito-Borne Microparasite Infections in Humans

**DOI:** 10.1155/2013/659038

**Published:** 2013-12-24

**Authors:** Marcos Amaku, Marcelo Nascimento Burattini, Francisco Antonio Bezerra Coutinho, Luis Fernandez Lopez, Eduardo Massad

**Affiliations:** ^1^School of Veterinary Medicine, University of São Paulo, Avenida Prof. Dr. Orlando Marques de Paiva 87, Cep 05508-270 São Paulo, SP, Brazil; ^2^School of Medicine, University of Sao Paulo and LIM 01-HCFMUSP, Rua Teodoro Sampaio 115, Cep 05405-000 São Paulo, SP, Brazil; ^3^CIARA, Florida International University, Miami, FL 33199, USA; ^4^London School of Hygiene and Tropical Medicine, London University, London WC1E 7HT, UK

## Abstract

To determine the maximum equilibrium prevalence of mosquito-borne microparasitic infections, this paper proposes a general model for vector-borne infections which is flexible enough to comprise the dynamics of a great number of the known diseases transmitted by arthropods. From equilibrium analysis, we determined the number of infected vectors as an explicit function of the model's parameters and the prevalence of infection in the hosts. From the analysis, it is also possible to derive the basic reproduction number and the equilibrium force of infection as a function of those parameters and variables. From the force of infection, we were able to conclude that, depending on the disease's structure and the model's parameters, there is a maximum value of equilibrium prevalence for each of the mosquito-borne microparasitic infections. The analysis is exemplified by the cases of malaria and dengue fever. With the values of the parameters chosen to illustrate those calculations, the maximum equilibrium prevalence found was 31% and 0.02% for malaria and dengue, respectively. The equilibrium analysis demonstrated that there is a maximum prevalence for the mosquito-borne microparasitic infections.

## 1. Introduction

Vector-borne diseases such as malaria, dengue, yellow fever, plague, trypanosomiasis, and leishmaniasis have been major causes of morbidity and mortality through human history [1].

Currently, half of the world's population is infected with at least one type of vector-borne pathogens [2, 3]. Only one mosquito-borne infection, dengue fever, affects the lives of 3.6 billion people worldwide [4, 5].

In the 17th through early 20th centuries, human morbidity and mortality due to vector-borne diseases outstripped all other causes combined [6]. By the 1960s the majority of vector-borne infections have been effectively controlled or targeted for intensive programmes. However, such programmes were discontinued in the 1970s because vector-borne infections were no longer considered major public health problems [7–10]. As a consequence, in the 1980s, the world observed a resurgence of old vector-borne diseases and the emergence of new ones [11].

The historical paradigm of mosquito-borne infections, malaria, accounts for the most deaths than any other human vector-borne diseases, with approximately 300 million people infected and up to one million deaths every year [12, 13]. Explosive epidemics have also marked the resurgence of dengue and yellow fever [1], and a great number of the most important vector-borne human diseases have exhibited dramatic changes in incidence and geographic range in recent decades [11].

Vectors of human diseases are typically species of mosquitoes that are able to transmit viruses, bacteria, or parasites to humans and other warm-blooded hosts [14]. Among the mosquito-borne infections, arthropod-borne viruses (arboviruses) comprise the largest class of vector-borne human pathogens with more than 500 arboviruses being described up to now, 20 percent of which causing human diseases [6, 15, 16]. Examples of arboviruses include dengue and dengue haemorrhagic fever, yellow fever, Rift Valley fever, West Nile virus, and Japanese encephalitis among [6, 16, 17].

Approximately 80 percent of vector-borne disease transmission typically occurs among 20 percent of the host populations [19, 20]. Thus, the overwhelming impact of the distribution of vector-borne infections is disproportionately on tropical and subtropical countries [2]. Unfortunately, this considerable economic, ecological, and public health impact of vector-borne infections is expected to continue, given limited capabilities for detecting, identifying, and addressing likely epidemics [1].

This paper proposes a general model for vector-borne infections which is flexible enough to comprise the dynamics of some known diseases transmitted by arthropods. From equilibrium analysis, we determined the number of infected vectors as an explicit function of the model's parameters and the prevalence of infection in the hosts. From the analysis, it is also possible to derive the basic reproduction number and the equilibrium force of infection as a function of those parameters and variables. From the force of infection, we were able to conclude that, depending on the disease's structure and the model's parameters, there is a maximum value of equilibrium prevalence for each mosquito-borne microparasitic infections. This is important because of the following: (a) knowing the maximum prevalence at equilibrium (neglecting seasonal variations, see below), we can calculate the maximum force of infection and, therefore, the maximum probability that a visitor gets the disease when visiting an endemic region; (b) the maximum force of infection can be immediately used to calculate the maximum incidence of the disease (again neglecting seasonal variations) in an affected endemic region; (c) if a vector-borne disease is introduced in an unaffected area, we can predict the maximum prevalence at equilibrium for the demographic and disease-related parameters in that area. This gives a very good idea of the amount of resources that will be needed to care for these cases. This allows public health authorities to anticipate the (economic and social) importance of a given disease in order to increase public health preparedness to deal with such challenges.

The model we present in this paper is a deterministic model. However, as we will show, it is possible to introduce some elements of stochasticity.

The whole analysis is exemplified by the cases of malaria and dengue fever.

## 2. The Model

The model that is used to calculate the efficiency of control strategies can be found in [21–23].

The populations involved in the transmission are human hosts, mosquitoes, and their eggs. For the purposes of this paper, the term “eggs” also includes the intermediate stages, such as larvae and pupae. Therefore, the population densities are divided into the compartments described in Table 1.

We first write down the model equations and then explain the meanings of their terms. The model equations are

(1) dSHdt=−abIMSHNH−μHSH+rHNH(1−NHκH)+σHRH+θHIH,dLHdt=abIMSHNH−(μH+δH)LH,dIHdt=δHLH−(μH+αH+γH+θH)IH,dRHdt=γHIH−μHRH−σHRH,dSMdt=pcS(t)SE−μMSM−acSMIHNH,dLMdt=acSMIHNH−γMLM−μMLM,dIMdt=γMLM−μMIM+pcS(t)IE,dSEdt=[rMSM+(1−g)rM(IM+LM)]×(1−(SE+IE)κE)−μESE−pcS(t)SE,dIEdt=[grM(IM+LM)](1−(SE+IE)κE)−μEIE−pcS(t)IE,where  NH=SH+LH+IH+RH,NM=SM+LM+IM,NE=SE+IE

and *c*_*S*_(*t*) = (*d*_1_ − *d*_2_sin(2*πft* + *ϕ*)) is a factor mimicking seasonal influences in the mosquito population [5, 24]. The seasonal influence was considered in another paper [21]. In this paper, however, as a first approximation, which is very good in some tropical areas, *c*_*s*_(*t*) = *c*_*s*_ = constant.

**Remark 1**

As mentioned above, seasonal influence in the mosquito population was not considered in this paper. The reason for this is that including seasonal variation, that is, considering *c*_*s*_(*t*) ≠ constant, implies additional analytical difficulties (see, e.g., [25–27]). In most tropical regions, the mosquito population varies very little along the year [28], and, therefore, this additional complication is unnecessary. Furthermore, at least for part of the year, the equilibrium is reached even when seasonality is important. Also, in this paper, we want to make a comparison of the maximum prevalence attained at endemic equilibrium among various vector-transmitted diseases. As explained in the Introduction, endemic equilibrium is important to help public health authorities to plan and control such diseases and to better understand the enormous differences in prevalence of distinct vector-borne diseases like the observed equilibrium prevalence of malaria as compared to dengue.

The model's parameters are described in Table 2.

Let us explain the meaning and limitations of the above model. First, as mentioned, the variables are densities, that is, number of humans/vectors per unit area. Therefore, to use the model as it is written above, we should consider an area where the populations are approximately homogenously distributed and multiply each variable by this area. One particular important point is raised by the term *abI*_*M*_(*S*_*H*_/*N*_*H*_).

Let us explain the meaning of this term. The parameter *a* is a composed quantity. Let *A* be the area explored by a mosquito by the joint movement of the humans and the mosquitoes. Let *ξ* be the number of bites a mosquito inflicts per unit time and per unit area in the humans. Then, *ξAI*_*M*_ is the number of bites that *AI*_*M*_ infected mosquitoes inflict on *N*_*H*_ *A* people. Hence, the fraction of bites given on susceptible humans is *ξAI*_*M*_(*S*_*H*_ *A*/*N*_*H*_ *A*) = *aI*_*M*_(*S*_*H*_/*N*_*H*_), where *a* = *ξA*.

Therefore, the number of susceptible humans that get the infection per unit time from infected mosquitoes is *abI*_*M*_(*S*_*H*_/*N*_*H*_), where *b* is the probability that a bite from an infected mosquito results in an infected (latent) human.

Analogously, the term *ac*(*I*_*H*_/*N*_*H*_)*S*_*M*_ represents the density of new infections per unit time in mosquitoes due to mosquitoes' bites on infective humans.

The term *r*_*H*_ *N*_*H*_(1 − *N*_*H*_/*K*_*H*_), where *r*_*H*_ is the Malthusian parameter and *K*_*H*_ is the carrying capacity, represents the birth rate per unit area. We assume that all the individuals are born susceptible.

The terms

(2) $$\begin{array}{c} \left[r_{M}S_{M}+\left(1-g\right)r_{M}\left(I_{M}+L_{M}\right)\right]\left(1-\frac{\left(S_{E}+I_{E}\right)}{\kappa_{E}}\right),\\ \left[gr_{M}\left(I_{M}+L_{M}\right)\right]\left(1-\frac{\left(S_{E}+I_{E}\right)}{\kappa_{E}}\right) \end{array}$$

represent the birth rate per unit area of susceptible and infected eggs, respectively. Note that we assume that eggs can be born infected, a phenomenon called vertical transmission in the literature.

The term *pc*_*s*_(*t*)*S*_*E*_ represents the number of noninfected eggs per unit time per unit area that reaches the adult stage. The parameter *c*_*s*_(*t*) was introduced to mimic seasonality and, as mentioned before, in this paper is made constant. The term *pc*_*s*_(*t*)*I*_*E*_ represents the number of infected eggs per unit time per unit area that reaches the infected adult stage.

The other terms are transition terms between the compartments as explained, for example, in [22].

Model (1) is a very general model for some known vector-borne infections. Therefore, depending on the values of some parameters, the model can describe any type of dynamics in the human subpopulation, such as that seen in Table 3.

The model can also include vaccination, for instance, against yellow fever or dengue, but this subject will not be treated in this work.

From system (1), it is possible to determine the equilibrium densities of the variables of interest. We carried out detailed equilibrium analysis in a related article [18]. For our purposes, we calculate the equilibrium densities of *I*_M_*, the number of infected mosquitoes:

(3) IM∗=(δH+μH)(μH+γH+αH+σH)IH∗ ×(abδH  ×(1−((μH+σH)(μH+γH+αH+δH+θH)+γHδHδH(μH+σH))  ×IH∗NH∗))−1.

**Figure 1:**
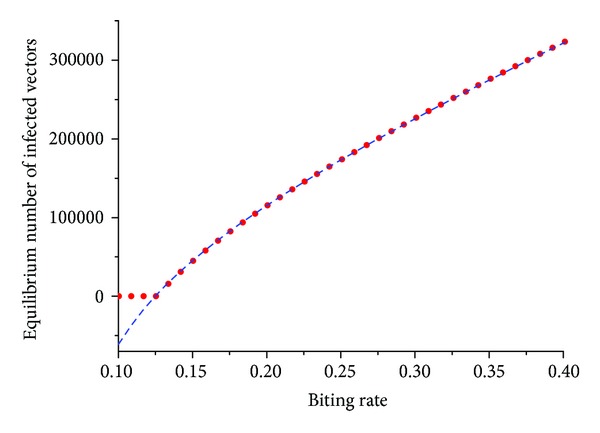
Plot of *I*_M_* (3) as a function of the biting rate, *a*, calculated in two ways: (a) the red dotted line shows the number of infected vectors calculated with the host prevalence *I*_H_*/*N*_H_* directly derived from the numerical solution of the dynamics of system (1); (b) the blue dashed line shows the number of infected vectors calculated with the host prevalence *I*_H_*/*N*_H_* derived from (4). For *a* such that *R*_0_ is less than one, we have *I*_H_*/*N*_H_* < 0. For *a* such that *R*_0_ is greater than one, the two curves coincide.

Replacing the values of *I*_H_* and *N*_H_* given in (3), it is possible to see that *I*_M_* increases with the biting rate *a*, as shown in Figure 1.

The expressions for *I*_H_* and *N*_H_*, in terms of the model's parameters, that appear in (3) are

(4) IH∗NH∗ =(γM+gμM)a2bc(NM∗/NH∗)−Q(μM+γM)μM(1−g)(γM+gμM)a2bcδH(NM∗/NH∗)Z+acQ(μM+γM),

which is the equilibrium prevalence of the infection in humans and where

(5) $$\begin{array}{c} Q=\left(\frac{\left(\mu_{H}+\sigma_{H}\right)\left(\mu_{H}+\gamma_{H}+\alpha_{H}+\delta_{H}+\theta_{H}\right)+\gamma_{H}\delta_{H}}{\delta_{H}\left(\mu_{H}+\sigma_{H}\right)}\right),\\ Z=\left[\frac{\left(\left(\delta_{H}+\mu_{H}\right)\left(\mu_{H}+\gamma_{H}+\alpha_{H}+\sigma_{H}\right)\right)}{\delta_{H}}\right],\\ N_{M}^{*}=\frac{pc_{S}}{\mu_{M}}\kappa_{E}\left[1-\frac{\left(\mu_{M}\right)\left(\mu_{E}+pc_{S}\right)}{r_{M}pc_{S}}\right]. \end{array}$$

The calculation of the total human population expression at equilibrium, *N*_H_*, is slightly more complicated and results in

(6) $$\begin{array}{c} N_{H}^{*}=\frac{-B+\sqrt{B^{2}-4AC}}{2A}, \end{array}$$

where

(7) $$\begin{array}{c} A=acr_{H}\Omega ,\\ B=-ac\Omega \kappa_{H}\left(r_{H}-\mu_{H}\right)+\Gamma Zr_{H}-\Omega \mu_{M}\left(1-g\right)\alpha_{H}\kappa_{H},\\ C=-\Gamma \kappa_{H}\left(r_{H}-\mu_{H}\right)Z+\Gamma \alpha_{H}\kappa_{H},\\ \Omega =Q\left(\gamma_{M}+\mu_{M}\right),\\ \Gamma =\left(\gamma_{M}+g\mu_{M}\right)a^{2}bc\delta_{H}N_{M}^{*\;\;}. \end{array}$$

From (4), it is possible to deduce the expression of the basic reproduction number of model (1) as [22, 29, 30]

(8) R0=(γM+gμM)a2bcNM(0)NH(0)  ×((μH+σH)(μH+γH+αH+δH+θH)+γHδHδH(μH+σH)    ×(μM+γM)μM(1−g))−1,

where *N*_*M*_(0) and *N*_*H*_(0) are the population of vectors and hosts calculated in the absence of the infection.

Figure 1 is a plot of (3) as a function of the biting rate, *a*, calculated in two ways: in the red dotted line the number of infected vectors is calculated with the host prevalence *I*_H_*/*N*_H_* directly derived from the dynamics of system (1); in the blue dashed line *I*_H_*/*N*_H_* is calculated from (4) and for *a* such that *R*_0_ can be less than one and, therefore, *I*_H_*/*N*_H_* < 0.

Note that, as expected, the number of infected vectors is a monotonically increasing function of the biting rate.

The force of infection (the incidence density rate) for humans at the equilibrium, *λ*_H_*, is defined as

(9) $$\begin{array}{c} \lambda_{H}^{*}=ab\frac{I_{M}^{*}}{N_{H}^{*}}, \end{array}$$

which can be explicitly written in terms of the equilibrium prevalence of the infection in humans (3) as

(10) λH∗=(δH+μH)(μH+γH+αH+σH)IH∗NH∗ ×(δH×(1−((μH+σH)(μH+γH+αH+δH+θH)+γHδHδH(μH+σH))×IH∗NH∗))−1.

From (10), it can be seen that the equilibrium prevalence of infection among humans, *I*_H_*/*N*_H_*, is less than a certain value:

(11) (IH∗NH∗) <δH(μH+σH)(μH+σH)(μH+γH+αH+δH+θH)+γHδH.

**Figure 2:**
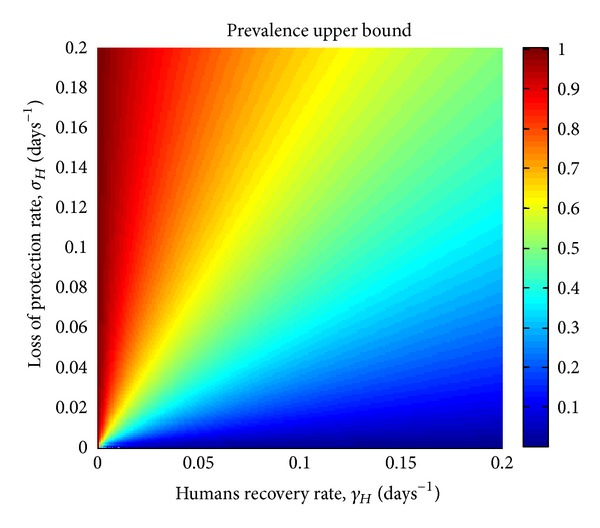
Some theoretical vector-borne infections and their maximum equilibrium prevalences in humans as a function of the recovery rate, *γ*_*H*_, and the rate of loss of immune protection, *σ*_*H*_. The values of the other parameters are: *μ*_*H*_ = 4.57 × 10^−5^ days^−1^, *δ*_*H*_ = 0.1 days^−1^, *α*_*H*_ = 0.01 days^−1^, and *θ*_*H*_ = 0.

Hence, depending on the disease, the model's structure will determine whether the maximum equilibrium prevalence

(12) $$\begin{array}{c} {\left(\frac{I_{H}^{*}}{N_{H}^{*}}\right)}_{\text{MAX}}=\frac{\delta_{H}\left(\mu_{H}+\sigma_{H}\right)}{\left(\mu_{H}+\sigma_{H}\right)\left(\mu_{H}+\gamma_{H}+\alpha_{H}+\delta_{H}+\theta_{H}\right)+\gamma_{H}\delta_{H}} \end{array}$$

is large or small. For instance, in a SEIR model such as dengue, where the recovery rate, *γ*_*H*_, is large relatively to the human mortality rate, *μ*_*H*_, the maximum equilibrium prevalence of the infection in humans is very low.  Figure 2 exemplifies some theoretical vector-borne infections and their maximum equilibrium prevalence in humans as a function of the recovery rate, *γ*_*H*_, and the rate of loss of immune protection, *σ*_*H*_.

Let us illustrate the theory above by comparing two very distinct vector-borne infections, namely, malaria and dengue. In Table 4 we show the typical values of the key parameters that determine the maximum equilibrium prevalence for both malaria and dengue, as in (11).

By applying the parameters' values above to (12) we end up with maximum equilibrium prevalences of 31% for malaria and 0.02% for dengue, which are in accord with typical prevalences found in endemic places for both diseases. A summary of the sensitivity analysis of the model is presented in the Appendix. This sensitivity analysis was carried out on the parameters that are related to dengue control.

Finally, a comment on an important aspect of (8) for the basic reproduction number, *R*_0_. This expression has a discontinuity when *g* = 1; that is, when 100% of the eggs are laid infected. This is a theoretical possibility and when *g* → 1, there is a structural change in our model. The populations of susceptible and infected eggs become completely decoupled. It can be verified that the disease is able to sustain itself even without human hosts. As a matter of fact, as previously demonstrated [31], this is the only way the infection circulates exclusively among vectors without the hosts.

In addition, when *g* = 1 and the human hosts are introduced into the system, then since all the eggs of infected mosquitoes are infected, the time evolution leads to a situation where all mosquitoes are infected. Therefore, when *g* = 1 and human hosts are introduced, the population of susceptible mosquitoes and eggs goes to zero. In any case, there is no known infection that is 100% transmitted to mosquitoes' eggs.

## 3. Discussion

Since the seminal work by Ronald Ross, mathematical models have provided a great deal of theoretical support for understanding the complex dynamics of vector-borne infections, in addition to the important role those models have played in designing and assessing control strategies [32]. Key concepts like the basic reproduction number, vectorial capacity and the force of infection derived from the theoretical works on vector-borne infections are currently central to the quantification of transmission, as well as to the proposal of public health measures to control them [22].

In this work, we propose a general, although very sketchy, model that considers a great deal of the aspects related to the dynamics of mosquito-borne microparasites. From the equilibrium analysis, we calculated the prevalence of the infection in the host populations, from which the number of infected vectors was deduced. In addition, we deduced an explicit expression for the basic reproduction number and the equilibrium force of infection. It was possible then to demonstrate that, provided an equilibrium is reached, each mosquito-borne microparasite has a maximum host prevalence, depending on the disease's structure and on the value of the parameters. This analysis was exemplified by the calculation of the maximum equilibrium prevalence of malaria and dengue. Once the disease's structure is determined and the values of the parameters are known, it is possible to calculate the maximum equilibrium prevalence of a mosquito-borne microparasitic infection.

It may be argued that malaria is not exactly a good example of a microparasitic infection. However, although malaria can behave sometimes as a microparasite and sometimes as a macroparasite [33], in the specific context of the proposed model, it can be considered as a microparasitic disease.

Another important limitation of our approach is that, in order to calculate the equilibrium densities of each of the model's variables, we have to neglect seasonal fluctuations, which can be very important in the transmission dynamics of such infections like dengue. However, seasonality in some tropical areas is not too important and the results can be applied to the average trend in prevalence levels.

At first inspection, (3) may seem odd because the biting rate appears in the denominator and the number of infected mosquitoes should be directly proportional to that rate. However, if we write (3) as a function of the parameters only, that is, by writing the human prevalence term *I*_H_*/*N*_H_* as an explicit function of the model's parameters, it is possible to see that the number of infected mosquitoes is indeed a monotonically growing function of the biting rate, as shown in Figure 1. We do not show this full equation because it is awkwardly big.

The calculation of a maximum value of equilibrium prevalence for a mosquito-borne microparasitic infection may help public health authorities to estimate the resources needed to care for the infected individuals, anticipating the importance of the disease and increasing the public health preparedness to deal with such a challenge.

## Figures and Tables

**Table 1:** Model variables and their biological meanings.

Variable	Biological meaning
*S*_*H*_	Susceptible humans density
*L*_*H*_	Latent humans density
*I*_*H*_	Infectious humans density
*R*_*H*_	Recovered humans density
*S*_*M*_	Uninfected mosquitoes density
*L*_*M*_	Latent mosquitoes density
*I*_*M*_	Infectious mosquitoes density
*S*_*E*_	Uninfected eggs (imm. stages) density
*I*_*E*_	Infected aquatic forms density

**Table 2:** Model's parameters and their biological significance.

Parameter	Biological meaning
*a *	Average daily rate of biting (see text)
*b *	Fraction of bites actually infective to humans
*σ*_*H*_	Loss of immunity rate
*δ*_*H*_	Latency rate in humans
*θ*_*H*_	Loss of infectiousness in humans
*μ*_*H*_	Human natural mortality rate
*r*_*H*_	Birth rate of humans
*κ*_*H*_	Carrying capacity of humans
*α*_*H*_	Disease mortality in humans
*γ*_*H*_	Human recovery rate
*p *	Hatching rate of susceptible eggs
*γ*_*M*_	Latency rate in mosquitoes
*μ*_*M*_	Natural mortality rate of mosquitoes
*r*_*M*_	Oviposition rate
*g *	Proportion of infected eggs
*κ*_*E*_	Carrying capacity of eggs
*μ*_*E*_	Natural mortality rate of eggs
*c *	Fraction of bites actually infective to mosquitoes
*c*_*S*_	Climatic factor

**Table 3:** Model's structure as a function of the parameters.

Model's structure	*δ*_*H*_	*γ*_*H*_	*σ*_*H*_	*θ*_*H*_
SI	→ *∞*	0	0	0
SIS	→ *∞*	0	0	*≠*0
SIR	→ *∞*	*≠*0	0	0
SIRS	→ *∞*	*≠*0	*≠*0	0
SEIR	*≠*0	*≠*0	0	0
SEIRS	*≠*0	*≠*0	*≠*0	0

**Table 4:** Parameters' values that determine the maximum equilibrium prevalences of Malaria and Dengue.

Parameter	Malaria	Dengue
*σ*_*H*_	0.10 days^−1^	0.00 days^−1^
*δ*_*H*_	0.07 days^−1^	0.14 days^−1^
*θ*_*H*_	0.00 days^−1^	0.00 days^−1^
*μ*_*H*_	4.57 × 10^−5^ days^−1^	4.57 × 10^−5^ days^−1^
*α*_*H*_	10^−3 ^ days^−1^	10^−5 ^ days^−1^
*γ*_*H*_	0.14 days^−1^	0.20 days^−1^

**Table 5:** Results of the sensitivity analysis. The results represent the relative amount of variation (expressed in percentual variation) in the variable if we vary the parameters by 1% (see [18] for details).

Parameter	Mean
Sensitivity of *R*_0_ to the control parameters	
*a*	1.94
κ_*E*_	0.69
μ_*E*_	(−) 8.28 × 10^−4^
μ_*M*_	(−) 2.42

Sensitivity of λ to the control parameters	
*a*	5.02
*κ*_*E*_	2.32
μ_*E*_	(−) 1.93 × 10^−3^
μ_*M*_	(−) 5.40

Sensitivity of *I*_*H*_/*N*_*H*_ to the control parameters	
*a*	2.67
κ_*E*_	1.34
μ_*E*_	(−) 2.31 × 10^−2^
μ_*M*_	(−) 3.20

## Appendix

### Sensitivity Analysis to Some Parameters Related to Control

In this appendix, we present a summary of the sensitivity analysis (explored in detail in [18]) of the equilibrium results to some parameters related to control for the case of dengue. The analyzed variables are the basic reproductive number, the force of infection (incidence density), and prevalence. The parameters are the biting rate, *a*; the immature stages carrying capacity, *κ*_*E*_; the mortality rate of the larvae, *μ*_*E*_; and the mosquito mortality rate, *μ*_*M*_. These parameters correspond to the actual control strategies available. The results are shown in Table 5.
